# Supplementary material for: Hunger shifts attention and attribute weighting in dietary choice
Source: eLife. 2025 Jul 2;13:RP103736. doi: 10.7554/eLife.103736 (PMC12221300; doi:10.7554/eLife.103736)
Supplement: Figure 2—figure supplement 2—source data 1. [file elife-103736-fig2-figsupp2-data1.docx]

**Figure 2 – Figure Supplement 2**

*Factor Loadings on the Components for the Respective Datasets*

|  | Dim.1 | Dim.2 | Dim.3 | Dim.4 | Dim.5 |
| --- | --- | --- | --- | --- | --- |
| health_vd | 0.91 | -0.015 | -0.012 | 0.327 | 0.055 |
| taste_vd | 0.232 | 0.849 | -0.473 | -0.004 | 0.003 |
| nutri_diff | 0.907 | -0.188 | 0.021 | 0.067 | 0.268 |
| want_vd | 0.259 | 0.837 | 0.481 | -0.031 | 0.003 |
| cal_vd | -0.905 | 0.138 | -0.009 | -0.03 | 0.382 |
| total_cal_vd | -0.882 | 0.119 | 0.035 | 0.427 | -0.058 |

*Note*. vd refers to value difference left – right option.
