# Supplementary material for: Hunger shifts attention and attribute weighting in dietary choice
Source: eLife. 2025 Jul 2;13:RP103736. doi: 10.7554/eLife.103736 (PMC12221300; doi:10.7554/eLife.103736)
Supplement: Figure 3—figure supplement 2—source data 1. [file elife-103736-fig3-figsupp2-data1.docx]

**Figure 3 – Figure Supplement 2**

*Mediation coefficients*

|  | Mean | SE | Median | 2.50% | 97.50% | n_eff | Rhat |
| --- | --- | --- | --- | --- | --- | --- | --- |
| a | 0.01 | 0.01 | 0.01 | >0.001 | 0.02 | 10799 | 1 |
| b | 5.45 | 0.46 | 5.44 | 4.56 | 6.35 | 4233 | 1 |
| cp | 0.19 | 0.11 | 0.19 | -0.02 | 0.4 | 7101 | 1 |
| me | 0.09 | 0.04 | 0.09 | 0.02 | 0.16 | 9671 | 1 |
| c | 0.28 | 0.12 | 0.28 | 0.04 | 0.52 | 6852 | 1 |
| pme | 0.31 | 3.8 | 0.31 | 0.07 | 0.95 | 19812 | 1 |

*Note. a* is the effect of hunger state to attention, *b* is the effect from attention to choice, *cp* is the indirect effect of hunger state on choice taking attention into account, *c* is the direct effect of hunger state on choice, when not considering attention, *me* refers to the mediation effect, thus the combination of paths *a* and *b*, *pme* refers to the proportion of the effect that is mediated. Output refers to posterior, mean, standard deviation (=standard error; SE), median, and credible interval respectively. n_eff refers to the number of effective posterior samples, to obtain confident estimates it is recommended to be >100 Vuorre and Bolger (2018); R-hat is the scale reduction factor, to accurately predict posterior distributions, it should be 1.00, according to Vuorre and Bolger (2018) values within .05 are acceptable.
