# Supplementary material for: Hunger shifts attention and attribute weighting in dietary choice
Source: eLife. 2025 Jul 2;13:RP103736. doi: 10.7554/eLife.103736 (PMC12221300; doi:10.7554/eLife.103736)
Supplement: Figure 3—figure supplement 3—source data 1. [file elife-103736-fig3-figsupp3-data1.docx]

**Figure 3 – Figure Supplement 3**

*Standard deviations of subject-level effects (random effects), their covariances and correlations*

|  | Mean | SE | Median | 2.50% | 97.50% | n_eff | Rhat |
| --- | --- | --- | --- | --- | --- | --- | --- |
| tau_a | 0.03 | 0.01 | 0.03 | 0.02 | 0.04 | 8155 | 1 |
| tau_b | 3.53 | 0.36 | 3.5 | 2.89 | 4.29 | 5933 | 1 |
| tau_cp | 0.78 | 0.09 | 0.78 | 0.62 | 0.97 | 8300 | 1 |
| covab | 0.02 | 0.02 | 0.02 | -0.01 | 0.06 | 10451 | 1 |
| corrab | 0.21 | 0.15 | 0.21 | -0.1 | 0.5 | 10935 | 1 |
