# Supplementary material for: Hunger shifts attention and attribute weighting in dietary choice
Source: eLife. 2025 Jul 2;13:RP103736. doi: 10.7554/eLife.103736 (PMC12221300; doi:10.7554/eLife.103736)
Supplement: Supplementary file 1. [file elife-103736-supp1.docx]

**Appendix 13. General Participant Information About the Study**

**Title of the Study: The Effect of Hunger on Decision-Making Processes**

Welcome to our study on "The Effect of Hunger on Decision-Making Processes”! We appreciate your interest in participating. In this study, we are investigating how hunger affects value-based decisions, and the cognitive mechanisms involved.

**Study Procedure**

The experiment consists of two sessions. On one day, you will complete the experiment in a hungry state, and on another day, you will do so in a satiated state. Each session lasts approximately two hours. You will be asked to arrive at the lab in a fasted state for both sessions. In one of the sessions, you will receive a breakfast in the form of a protein shake tailored to your body size, weight, and gender. Apart from this breakfast, the rest of the procedure for both sessions are identical.

In the first part of the study, you will be asked to evaluate different options. In the second part, you will choose between pairs of options.

The evaluation tasks are conducted on a computer and include a food rating task, a social preference rating task and an intemporal discounting rating task. With respect to the food rating task, photos of actual, familiar foods served on plates. You will rate these with respect to liking, wanting, healthiness, and caloric content. In the social preference rating task, you will rate a proposed distribution of money between yourself and an NGO of your choice, represented abstractly by (partially filled) rectangles. A filled rectangle represents €10, an empty one represents €0. In the rating task of intertemporal discounting, you will rate proposals for receiving vouchers that differ in value and the timing of reception. The options are again represented abstractly by (partially filled) rectangles. The value is represented by the a horizontally filled rectangle (a filled rectangle represents €20, while an empty one represents €0). Timing is represented by a vertically filled rectangle (with a filled rectangle representing a 6-month delay and an empty one representing immediate delivery).

You will be asked to make value-based decisions by selecting one of two options. These options will involve the foods, social preference, and intertemporal discounting options you previously evaluated. The tasks will be conducted on a computer, and your eye movements will be recorded using an eye-tracking device. You will rest your head on a chinrest during these tasks to ensure accurate measurements. Additionally, the eye-tracking device will be calibrated individually to your eyes.

At the end of the experiment, one food item (food choice task), and either the money distribution for yourself and the charity (social choice task) or the voucher (intertemporal discounting task), will be randomly selected by a computer algorithm based on your choices. This item and/or amount will be given to you as a bonus. Therefore, you should carefully consider your choices during the tasks. You will receive the bonus at the end of the experiment (eventually, the voucher must be shipped per mail).

You will also be asked to rate your hunger, satiety and your current mood at various points using a visual analog scale and a brief questionnaire.

Finally, you will complete a questionnaire about your eating behavior and attitudes.

If you have any further questions, please ask the study supervisor.

**Voluntary Participation and Anonymity**

Participation in the study is voluntary. You may end your participation in this study at any time and without providing a reason, without any disadvantages resulting from it. Even if you withdraw early, you are entitled to appropriate compensation or credit for participant hours based on the time spent up to that point.

The data and personal information described above that are collected as part of this study will be treated confidentially. Project staff who have direct contact with your personal data are bound by confidentiality. Furthermore, the results of the study will be published in anonymized form, meaning your data cannot be linked to your identity.

**Data Protection**

The collection and processing of your personal data described above is carried out in a pseudonymized manner within the General Psychology department (Institute of Psychology at the University of Hamburg) using a number and without your name. There is a paper coding list that links your name with the number. This coding list is accessible only to the study supervisor and the project leader; this means only these individuals can associate the collected data with your name. The coding list is stored in a locked cabinet and will be destroyed after the data analysis is completed, but no later than October 1, 2025. After that, your data will be anonymized, making it impossible to link the data to your name. The anonymized data will be stored for at least 10 years.

As long as the coding list exists, you can request the deletion of all data collected about you. However, once the coding list has been destroyed, we will no longer be able to identify your dataset. Therefore, we can only comply with your request for data deletion while the coding list still exists.

**Scope of Data Collection and Processing**

The data may only be used for the investigation of decision-making processes and the cognitive processes involved.

**Collected Data:** Date of birth, gender, height, weight, handedness, educational level, occupation, monthly income, native language, and menstrual cycle (if applicable); hunger/satiety levels and mood; responses to rating and choice tasks; eye movements during choice tasks. Additionally, data such as your name, address, and phone number will be stored until anonymization. This is necessary to allow for the possibility of data deletion. These personal data will be kept securely and will not be made accessible to third parties.

**Analysis Results of the Data:** The collected data will be analyzed solely regarding decision-making processes and the cognitive processes involved. In accordance with the principles of good scientific practice, the data will also be made available to other researchers in an anonymized form.

**Retention Period for the Anonymized Data**

The fully anonymized data will be made publicly accessible via the Open Science Framework (www.osf.io). This will occur in an anonymized form, meaning the data cannot be linked to any specific person. This study follows the recommendations of the German Research Foundation (DFG) and the German Psychological Society (DGPs) for quality assurance in research. This approach ensures good scientific practice by allowing other researchers to verify the analysis or test alternative analyses.

**Your Rights**

In accordance with Article 13, Paragraph 2(b) of the General Data Protection Regulation (GDPR), you have the following rights:

1. **Right to Access (Art. 15 GDPR and §34 BDSG)**: You have the right to request information about the data processed about you and the potential recipients of this data at any time. You are entitled to a response within one month of submitting your request.
2. **Right to Rectification, Erasure, and Restriction (Art. 16-18 GDPR and §35 BDSG)**: You can request the rectification, erasure, or restriction of the processing of your personal data from the University of Hamburg at any time, as long as your data can still be associated with you (see above).
3. **Right to Data Portability (Art. 20 GDPR)**: You have the right to receive the personal data concerning you, which you provided to a data controller, in a structured, commonly used, and machine-readable format.
4. **Right to Object (Art. 21 GDPR and §36 BDSG)**: You have the right to withdraw your consent at any time with future effect. This can be done orally or by email. You may need to verify your identity. From the point the declaration is received, your data must no longer be processed and must be deleted immediately. Previous processing remains unaffected by this withdrawal.

If you wish to exercise any of these rights, please contact the project leader:

Jennifer March
Von-Melle-Park 11
20146 Hamburg
E-Mail: jennifer.march@uni-hamburg.de

Furthermore, you have the right to file a complaint with the supervisory authority:

The responsible state authority for data protection in Hamburg can be found via the service portal:<https://datenschutz-hamburg.de/>

Data Controller:

The President of the University of Hamburg
Mittelweg 177
20148 Hamburg
E-mail: [praesident@uni-hamburg.de](mailto:praesident@uni-hamburg.de)

Data Protection Officer:

Data Protection Officer of the University of Hamburg
Mittelweg 177
20148 Hamburg
E-mail: [datenschutz@uni-hamburg.de](mailto:datenschutz@uni-hamburg.de)

**Compensation**

You can choose between monetary compensation or course credit (the latter option is only available to psychology students at the University of Hamburg).

**For Monetary Compensation:** For participating in the study, you will receive a compensation of €25 (i.e., €12.50 per hour). The payment will be made in cash. Upon receiving the payment, you will be required to sign a receipt including your name. This information will be stored separately from the other data collected about you and serves as proof for potential expense audits. It will be deleted no later than October 1, 2025.

**For Course Credit:** Alternatively, you can opt to receive credit for participant hours equivalent to the time spent. For participating in this study, you will be credited with 2 participant hours.

Additionally, you will receive a food item and either money for yourself and the NGO or a voucher as a bonus.

If you end your participation early, you will receive proportional compensation for the time spent up to that point.
